# Whole-Genome Sequence of *Rummeliibacillus stabekisii* Strain PP9 Isolated from Antarctic Soil

**DOI:** 10.1128/genomeA.00416-16

**Published:** 2016-05-26

**Authors:** Fábio Faria da Mota, Renata Estebanez Vollú, Diogo Jurelevicius, Lucy Seldin

**Affiliations:** a Laboratório de Biologia Computacional e Sistemas, Instituto Oswaldo Cruz, Fundação Oswaldo Cruz (IOC/Fiocruz), Rio de Janeiro, Brazil; b Instituto de Microbiologia Paulo de Góes, Universidade Federal do Rio de Janeiro, Rio de Janeiro, Brazil

## Abstract

The whole genome of *Rummeliibacillus stabekisii* PP9, isolated from a soil sample from Antarctica, consists of a circular chromosome of 3,412,092 bp and a circular plasmid of 8,647 bp, with 3,244 protein-coding genes, 12 copies of the 16S-23S-5S rRNA operon, 101 tRNA genes, and 6 noncoding RNAs (ncRNAs).

## GENOME ANNOUNCEMENT

*Rummeliibacillus stabekisii* is constituted of strains of aerobic, Gram-positive, rod-shaped, round-spore-forming bacteria. Strains first described to belong to this species were isolated from different geographical locations (the type strain was isolated from the Payload Hazardous Servicing Facility at the Kennedy Space Center, FL), and their 16S rRNA gene sequence similarities demonstrated that they were most closely affiliated with *Bacillus pycnus* NRRL NRS-1691^T^ (98%), *Kurthia* sp. (96%), and *Viridibacillus* sp. (94 to 96%) (1). *R. stabekisii* was proposed as the type species of the genus *Rummeliibacillus* (1).

Strain PP9 was isolated from a soil sample collected from Punta Plaza located in King George Island, which is part of the South Shetlands archipelago in Maritime Antarctica, and identified as *R. stabekisii* (2). No genome sequences were available for any of the recognized species of the genus *Rummeliibacillus*. To gain a better understanding of this poorly studied species, the whole-genome sequence of *R. stabekisii* PP9 was determined.

The genome of *R. stabekisii* PP9 was sequenced in Seoul, South Korea, by DNA Link, Inc., using the PacBio RSII platform and two single-molecule real-time (SMRT) cells of P6-C4 chemistry with a 20-kb size-selected library; 450,876 raw reads resulted in 237,668 quality-filtered trimmed reads yielding 3,295 Mb, with a mean genome-wide coverage of about 969×. The filtered reads were assembled using HGAP version 2.3 (3). Annotation was performed using the NCBI Prokaryotic Genome Annotation Pipeline version 3.1 to predict protein-coding genes, structural RNAs, small noncoding RNAs, and tRNAs (4).

The genome of *R. stabekisii* PP9 consists of a circular chromosome of 3,412,092 bp and a circular plasmid of 8,647 bp, with a G+C content of 37.6%. The genome includes 3,244 protein-coding genes, 101 tRNA genes, 6 noncoding RNAs (ncRNAs), and 12 copies of the 16S-23S-5S rRNA operon. From the assigned protein-coding genes, only 1,939 have a putative function, while the remaining 1,305 genes (about 40%) were annotated as hypothetical proteins.

Genes related to sporulation and flagellar assembly were found in the PP9 genome. Round spores and motility observed by light microscopy and in soft agar stabbing, respectively, confirmed that their products are expressed and functional.

The average nucleotide identity (ANI) was calculated based on MUMmer (5) between the PP9 chromosome and six other complete chromosomes of strains belonging to the *Planococcaceae* family. *Kurthia* (accession no. CP013217.1) was the genus most closely related to *R. stabekisii* PP9 (accession no. CP014806.1), with 85.64% ANI, followed by *Solibacillus* (84.85%; accession no. NC_018065.1), *Jeotgalibacillus* (84.5%; accession no. CP009416.1), *Planococcus* (83.84%; accession no. CP013661.2), *Sporosarcina* (82.64%; accession no. CP014616.1), and *Planomicrobium* (82.45%; accession no. NZ_CCXS01000001.1). The resulting averages reflect a high degree of evolutionary distance between *R. stabekisii* and other members of its family.

### Nucleotide sequence accession numbers.

The genome sequences have been deposited at GenBank under the accession numbers CP014806 (chromosome) and CP014807 (plasmid). Strain PP9 is deposited in The Culture Collection of *Bacillus* and Related Genera (CCGB), under the accession no. CCGB1722.
